# DEMENTIA CARE IN ASIAN AND LATINO COMMUNITIES: PERSPECTIVES FROM FAMILY CAREGIVERS AND SERVICE PROFESSIONALS

**DOI:** 10.1093/geroni/igad104.3261

**Published:** 2023-12-21

**Authors:** Arienne Patano, Fei Sun, Daniel Vélez Ortiz, Ethan Liu

**Affiliations:** Michigan State University, East Lansing, Michigan, United States; Michigan State University, East Lansing, Michigan, United States; Michigan State University, East Lansing, Michigan, United States; Phillips Exeter Academy, Exeter, New Hampshire, United States

## Abstract

While community-based support and services are increasingly available to family caregivers (FCGs) of a person with dementia (PWD), those from ethnic minority groups tend to be underserved. This study aimed to identify unaddressed challenges to dementia care and uncover strategies to better assist FCGs within Asian and Latino communities in Michigan. Focus group and individual interviews were conducted with service professionals (SPs) and FCGs. Specifically, we ran two focus groups with SPs (n=11), and one with Asian FCGs (n=8). Four individual telephone interviews were conducted with Latino FCGs. Sessions were audiotaped and conducted in the participants’ preferred language, then transcribed and translated into English for analysis. Shared challenges to dementia care included: transportation to services; lack of translation services and language-concordant specialists, daycare/respite workers, and in-home care staff; and feelings of shame in needing support. Latino FCGs reported the emotional burden of witnessing the cognitive decline of their PWD, whereas Asian FCGs focused on challenges related to the behaviors of PWD. SPs and FCGs expressed the desire for more education for FCGs on dementia and self-management of stress. Limited technology literacy impacted their access to digital education. Culturally informed outreach and intervention strategies were recommended, such as partnering with community leaders and adapting cultural practices (e.g., providing family-oriented events, and using social media popular among specific ethnic groups). This study highlights the sociocultural challenges and practical outreach approaches for FCGs in Asian and Latino communities by integrating the perspectives of FCGs and SPs involved in dementia care.

